# Synchronous Squamous Cell Carcinoma in Multiple Digits

**Published:** 2011-02-23

**Authors:** Sabra Abner, Jeremiah Redstone, Saeed Chowdhry, Morton L. Kasdan, Bradon J. Wilhelmi

**Affiliations:** ^a^University of Louisville, Louisville, KY; ^b^Mt Sinai Medical Center, Chicago, IL

## Abstract

Cancers of the perionychium are relatively rare occurrences and are often related to chronic inflammation associated with trauma, infection, exposure to ultraviolet radiation, or other carcinogens. Squamous cell carcinoma is the most common tumor reported of the nail bed. Synchronous squamous cell carcinomas of the perionychium have been rarely reported. We present a case of a 46-year-old woman with synchronous squamous cell carcinomas involving both hands and multiple digits. Treatment modalities include chemotherapeutics, Mohs surgery, and amputation. Early diagnosis of squamous cell carcinoma of the nail bed provides the greatest chance to preserve maximal function of the hand. Onychomycosis may be the presenting symptom of a patient with squamous cell carcinoma and may also be a predisposing factor in patients with occupational risk factors. Suspicion of this disease process can help the clinician establish the diagnosis via biopsy and provide optimal care for these patients.

Cancers of the perionychium are relatively rare occurrences and are often related to chronic inflammation associated with trauma, infection, exposure to ultraviolet radiation, or other carcinogens. Squamous cell carcinoma is the most common tumor reported of the nail bed. Synchronous squamous cell carcinomas of the perionychium have been rarely reported. We present a case of a 46-year-old woman with synchronous squamous cell carcinomas involving both hands and multiple digits.

## CASE REPORT

**Figure 1:**
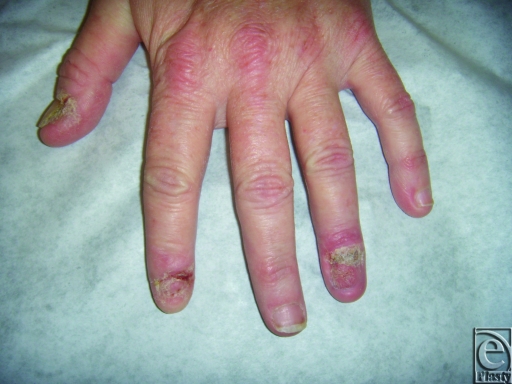
Photograph of 46-year-old women with multiple scaly lesions of both hands and multiple digits with biopsy-proven squamous carcinoma.

A 46-year-old white female was referred from the dermatology clinic with a biopsy-proven squamous cell carcinoma of the left ring finger perionychium. Physical examination demonstrated erythematous scaly lesions with the absence of a nail plate at the left thumb, index finger, and ring finger as well as the right long finger, without associated lymphadenopathy (Fig 1). She described chronic onychomycosis of the nails attributed to her job as a dishwasher.

Past medical history was significant for chronic onychomycosis. Social history demonstrated a 40 pack-year smoking habit. She denied alcohol use, exposure to aniline dyes, radiation, or arsenic exposure.

**Figure 2:**
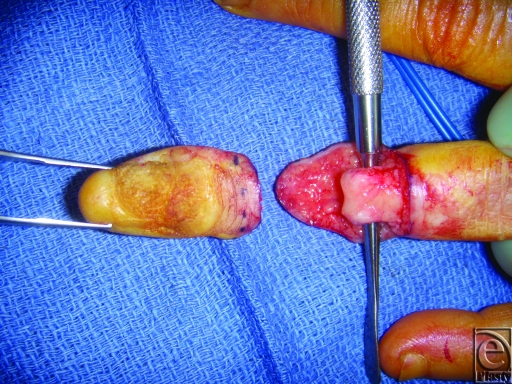
Amputation of the left index finger at the level of the distal interphalangeal joint.

The decision was made to biopsy all digits with scaly lesions. She was advised to stop smoking and counseled about the likely need for partial amputation. Pathology demonstrated squamous cell carcinoma of all biopsy sites. Wide local excision with partial amputation was performed on all involved digits with preservation of the left thumb interphalangeal joint and left index finger, left ring finger, and right long finger proximal interphalangeal (Fig 2).

**Figure 3:**
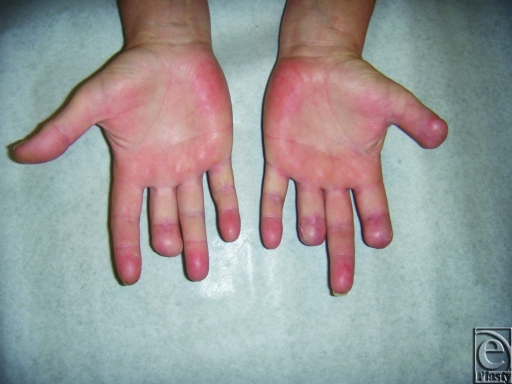
Healed wounds to bilateral hands.

Surgical pathology demonstrated positive margins on the left thumb and left ring finger necessitating reexcision. All final margins are clear and all incisions have healed nicely (Fig 3).

Due to the extensive nature of the patients' cancer and longstanding history, we elected to perform amputation as opposed to other treatment modalities, which will be described further in the discussion. The patient is currently about 1 year out from surgery and has returned to normal activities and displays no evidence of recurrence.

## DISCUSSION

Squamous cell carcinoma of the nail bed is more common in the 5th and 6th decades of life, most commonly involves the thumb, and has a male predominance.^1^ While the predisposing factors are not clear, it has been suggested that repeated trauma, chronic paronychia, and radiation may play a role^1^ as well as arsenic exposure and presence of human papillomavirus.^2^ Chronic paronychia is much more common in diabetic people, dishwashers, and maids.^3^

A review of the literature suggests that multiple digit involvement of the nail bed with squamous cell carcinoma is rare. In 1987, Baran and Gormley^4^ reviewed Bowen's disease in multiple digits and cited 5 previous cases as well as added 3 more to the literature. Five of the 8 cases reported involved both hands, and in 7 of the 8 cases, the patients were male. Trauma and radiation were suspected as predisposing factors in some cases. In 1995, a case report of a man with multiple digit involvement was reported where the predisposing factor was suspected to be trauma. Invasive squamous cell carcinoma was found only after amputation was performed.^5^ In 2007, a case was reported in a man with occupational exposure to stagnant wastewater and was subsequently found to have human papillomavirus positive lesions.^6^ These cases demonstrated the importance of occupational exposure in multiple digit involvement with squamous cell carcinoma.

Several treatment modalities for squamous cell carcinoma have been described. Treatment has consisted of 5-fluorouracil and a keratolytic ointment,^7^ Mohs micrographic surgery, skin and subcutaneous resection with 4 mm margins, or partial amputation. Most agree that maximum function should be preserved, and thus one author argues for Mohs micrographic surgery.^8^

Recurrence after Mohs surgery has been reported,^9^ and most authors believe amputation has the highest rate of cure. Indications for amputation have been described. When the carcinoma has been present for a long time, is large, or involves the bone, amputation at the distal interphalangeal or more proximally has been the routine treatment strategy.^2^

Early diagnosis of squamous cell carcinoma of the nail bed provides the greatest chance to preserve maximal function of the hand. Onychomycosis may be the presenting symptom of a patient with squamous cell carcinoma and may also be a predisposing factor in patients with occupational risk factors. Suspicion of this disease process can help the clinician establish the diagnosis via biopsy and provide optimal care for these patients.
